# Effect of Attentional Bias on the 3D Rotated Objects Recognition Ability of Dogs

**DOI:** 10.3390/ani13101673

**Published:** 2023-05-17

**Authors:** Marcello Siniscalchi, Serenella d’Ingeo, Angelo Quaranta

**Affiliations:** Animal Physiology and Behaviour Unit, Department of Veterinary Medicine, University of Bari Aldo Moro, 70121 Bari, Italy

**Keywords:** dog, cognition, physiology, visuo-spatial abilities, brain lateralization, mental rotation

## Abstract

**Simple Summary:**

The visual recognition of objects with different spatial orientations has been observed in the animal kingdom. This ability allows animals to adapt efficiently to a changing environment. A recent study indicated that dogs might be capable of recognizing rotated 2D objects, although differences between individuals were observed. To investigate dogs’ abilities to recognize rotated objects further, we trained six dogs to discriminate between 3D objects and their rotated versions (45° and 180°), which were presented on a computer screen. Our results revealed that dogs recognized three-dimensional objects and their rotated versions, and performed better when the target stimuli were presented in the left side of their attentional visual field, indicating the possible role of cerebral lateralization in mental rotation tasks.

**Abstract:**

The ability to recognize rotated objects has been widely reported in the animal kingdom. Studies on animal and human spatial cognition highlighted the importance of visuo-spatial cognitive capability for surviving in a dynamic world. Although domestic animals are frequently involved in activities requiring a high level of visuo-spatial ability, currently, little is known about their visuo-spatial skills. To investigate this issue, we trained six dogs to discriminate between 3D objects (using a modified version of the Shepard–Metzler task) that were then reproduced digitally on a computer. We found that the dogs recognized three-dimensional objects and their rotated versions (45° and 180°) more easily when presented on the left side of the screen, suggesting right hemisphere superiority in the control of visuo-spatial functions. Moreover, we report inter-individual variability in their performance in the visuo-spatial task. Our preliminary results suggest that dogs could use a rotational invariance process for the discrimination of 3D rotated shapes that deserves further investigation.

## 1. Introduction

The ability to recognize objects, regardless of their spatial orientation, is fundamental for surviving in a dynamic world [1]. Animal and human visuo-spatial cognitive capabilities have received increasing attention in recent years [2,3,4]. However, the mechanisms underlying spatial cognition remain the topic of ongoing debate. Although behavioural evidence demonstrates the ability to recognize rotated objects in humans [1,2,5,6], no consensus has been reached on the processes that allow this skill. Mental rotation has been reported for the discrimination of identical or mirror images of visual forms [2,7]. This cognitive ability implies the generation of a mental image that has then been rotated and compared with the visual stimulus [3]. More recently, it has been hypothesized that humans may rely on several processes for object recognition that could be related to a viewpoint frame of reference (as a collection of specific stored views) or could be independent of the viewpoint (i.e., the objects conserve their structural features across different views), or they may rely on a combination of both processes [1]. These complex cognitive mechanisms support the ability to recognize rotated objects. It is, however, dependent on orientation disparities in humans that indeed show lower accuracy and longer reaction times with increasing angle of stimuli rotation in visual discrimination tasks [2,8].

The visual recognition of objects with different spatial orientations has also been observed in the animal kingdom. Similar to humans, sea lions are capable of mentally rotating two-dimensional shapes. In support of the model of mental rotation, sea lions’ reaction times increased linearly with angular disparity [9]. Pigeons, meanwhile, show a different visual-information-processing system that is independent of the angle of rotation, a process known as rotational invariance [2]. Their reaction time to identify rotated objects was indeed not affected by the increase in the angle of rotation of the visual stimulus in a matching-to-sample task. This ability is crucial for efficient visual recognition of objects from a birds’-eye view, which operates on the horizontal plane during flight [2]; consequently, it favours birds’ orientation without any delay [9]. It has emerged that visuo-spatial cognition, and particularly the processes of visual information, are related to specific ecological demands. In species such as humans, whose visual systems primarily operate on the frontal plane, where an object’s orientation is highly consistent and dependent on gravity [2,9], distinct have strategies evolved (as reported above). Different visual information processing systems may also coexist in species with overlapping ecological demands, as reported for Rhesus monkeys [10]. In this terrestrial specie, two separate strategies were observed for the discrimination of rotated shapes. While a rotational invariance mode was clearly shown for small angles of rotation (0°–120°), different information processing, which potentially evolved as an intermediate between rotational invariance and mental rotation, has been reported for the highest angles (i.e., 160°) [10].

Besides species-specific traits, research on visuo-spatial cognitive abilities has revealed inter-individual variability that has been extensively explored in humans. Gender differences in mental rotation favouring males have been reported for 2D objects (but disappear when 3D objects are presented) [11,12]. The stability of such differences is challenged by training, which significantly affects mental rotation performance in women by increasing their accuracy to the level of that of men [13]. Moreover, everyday activities involving spatial demands significantly improve mental rotation performance [14], suggesting that this cognitive ability is rather malleable and could be influenced by life experience in using spatial abilities [6]. Individual differences in the strategy to recognise rotated objects could also be determined by the local or global processing of an object’s details, which cause a different focus of the subjects’ attention to the general feature of the object, or to specific and distinctive parts of it (local attention) [15]. 

Studies on the neural mechanisms that underlie mental rotation in humans have mainly focused on possible cerebral lateralization [16]. Although there is a great consensus regarding the right hemisphere specialization for spatial abilities, neurophysiological and lesion studies report ambiguous results on the brain hemisphere specialization for mental rotation [17], suggesting that this complex cognitive ability may rely on both hemispheres to contribute and cooperate [3]. Moreover, different and specific variables have been shown to influence the involvement of the right and the left hemispheres in processing rotated objects, including gender [16,17], individual spatial abilities [16,18], level of practice in mental rotation tasks [18,19], type of task (using 2D or 3D objects [18]) and handedness [5,17,20]. To date, evidence of brain lateralization for the recognition of rotated objects in animals is scarce. Preliminary studies have shown differences in the information-processing model used by baboons to solve a rotational task according to the visual hemifield involved: they displayed a human-like mental rotation when mirror-image shapes were in their right hemifield and a pigeon-like rotational invariance when shapes were in their left visual hemifield [21]. Considering that the stimuli presented in each visual hemifield were mainly analysed by the contralateral hemisphere [22], the left and the right hemispheres appeared to contribute to the parallel processing of visual stimuli with different, but potentially complementary, specializations [10].

In a recent study, the first evidence of dogs’ abilities to recognize two-dimensional rotated shapes was reported [23]. In this study, dogs were presented with a modified version of the Shepard–Metzler task [24] that consisted of the simultaneous presentation of two objects that differed in their spatial orientation or spatial configuration. The dogs were asked to indicate (by touching with their nose) which of the two objects was identical to the training one (previously associated with a reward), except for its spatial orientation. It was found that the spatial orientation had no effect on the dogs’ accuracies, but the non-rotated shapes were better recognized than the rotated stimuli. Individual variability was also reported, suggesting the existence of individual strategies for solving the visuo-spatial task or different skill levels. The mixed and complex nature of this study’s results raises questions about the informational processing mode used by dogs to process rotated visual stimuli and the factors that influence such cognitive ability. However, although there is evidence of the presence of specializations of the cerebral hemispheres of canines for spatial functions, to date, there are no data on the possible relationship between lateralization and image rotation in this species. Moreover, although previous studies have reported that dogs have the neuro-cognitive potential to recognize two-dimensional digital objects [23], evidence of dogs’ abilities to discriminate three-dimensional rotated objects, which better resemble natural objects, and the underlying cognitive processes is lacking. Considering that the task’s complexity (using 2D or 3D objects) affects the cognitive processes underlying the spatial abilities [18], we expected to observe potential differences in the dogs’ performances with respect to those observed for 2D objects. 

For the first time, we presented three-dimensional objects reproduced digitally on a computer screen to evaluate dogs’ abilities to discriminate their rotated versions; in addition, we investigated the possible effect of visual lateralization on the discrimination of three-dimensional presentations in a modified version of the Shepard–Metzler task.

## 2. Materials and Methods

### 2.1. Participants

The participants were comprised of 18 adult pet dogs and their owners, who voluntarily joined the experiment. All dogs underwent clinical evaluation at the Department of Veterinary Medicine, University of Bari, to assess the potential presence of vision impairments. After a behavioural evaluation was carried out by a veterinary behaviourist, six dogs were excluded from the study for their high arousal level registered in the experimental room that could significantly impact their ability to learn. The high arousal level was related to their fear of unknown humans and novel environments (3 dogs) and their excessive excitement (3 dogs). Two dogs were further excluded due to their low food motivation. Therefore, 10 subjects took part in the training procedure. Of these, four dogs abandoned the study due to the owners’ unavailability, which was mainly related to the COVID-19 pandemic. Hence, six dogs completed the training and underwent the 3D mental rotation task.

They were four females (three neutered) and two males (both neutered), whose ages ranged between 2 and 9 years (mean = 6.33; S.D. = 2.42). They were four mixed-breed dogs, one Border Collie and one Maltese. All of them were experimentally naïve and received no prior training. 

### 2.2. Stimuli

**Figure 1:**
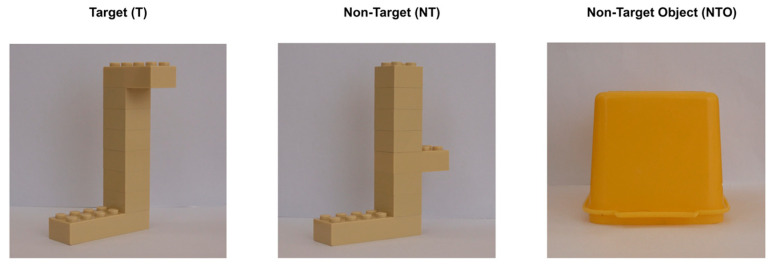
Three-dimensional real-life objects that were used for the “real-life” target and discrimination training.

A 3D real-life object was used to train the dogs to perform the task (see Section 2.4.1 Training). It consisted of eight yellow LEGO™ bricks (one 2 × 6 brick, one 2 × 4 brick and six 2 × 2 bricks) that were assembled to create a geometric shape (i.e., “target”, Figure 1). The target (T) was novel for all dogs, who had not seen it before the experiment. The yellow colour was selected as it falls within the visible spectrum of dogs [25]. 

Initially, the dogs were trained to recognize the target zero view (0°) and touch it with their nose (see the “Real-life” target training paragraph for the detailed procedure). In the following training stages (i.e., Discrimination training, see below), two yellow plastic boxes (non-target objects, NTO; Figure 1) were used to teach the dogs to discriminate the target (zero view) from other non-target objects. Once this ability was developed, the dogs were trained to discriminate the target from a similar visual stimulus (i.e., non-target, NT; Figure 1) made up of the same type and number of LEGO™ bricks, but with a different shape, both at zero view (0°).

**Figure 2:**
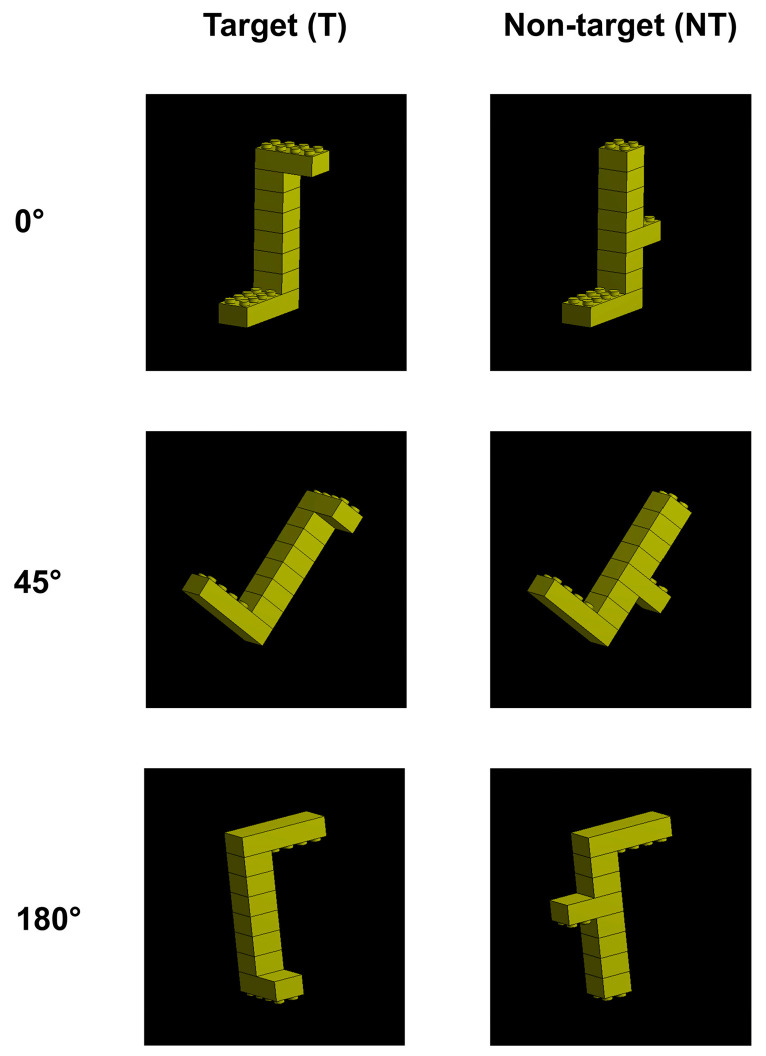
The digital target (T) and non-target (NT) used during the digital target training (zero view, 0°) and the 3D mental rotation task (0°, 45°, 180°).

For the digital target training and the 3D mental rotation task (see below for the detailed procedure), realistic digital representations of the T and NT were created using Ansys^®^ 3D design software (see Figure S1, Supplementary Materials). The use of digital images allowed for a standard and repeatable rotation of the objects that were presented during the 3D mental rotation task. Although dogs were trained to recognize or discriminate 2D digital visual stimuli directly on the screen in previous studies [26,27,28,29], a preliminary phase with real-life 3D objects was preferred because, currently, there is no evidence of dogs’ abilities to recognize 3D objects when presented digitally on a screen. This phase was then used to facilitate the subjects’ training. Three different 3D pictures for each object (T and NT) were obtained: the zero view (0°), which represents the T and NT with the spatial orientation used for the digital training stage, and the 45° clockwise rotation and the 180° rotation that were both employed for the 3D mental rotation task (Figure 2). As previous studies found no differences in dogs’ abilities to recognize 2D objects rotated clockwise and counterclockwise with different angles [23], only 45° clockwise rotations of T and NT were considered in this study.

Stimuli were displayed during the experiments as PowerPoint slideshows. They were presented on a uniform black background.

### 2.3. Experimental Setup

**Figure 3:**
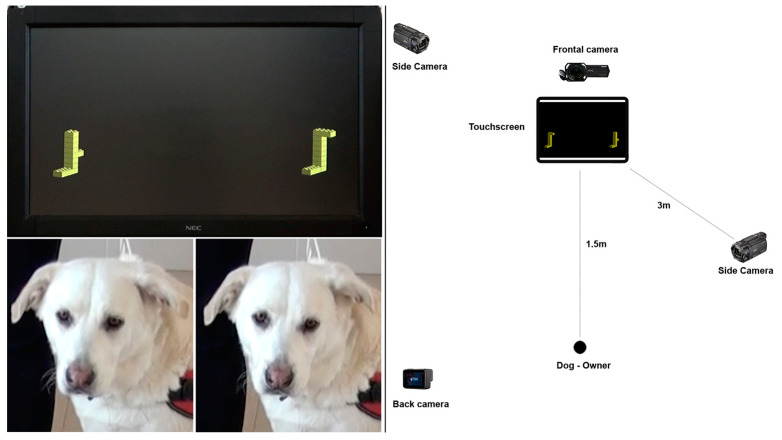
Testing apparatus of the digital target training and the 3D mental rotation task.

The experiment was carried out in an isolated room of the Department of Veterinary Medicine, University of Bari (5.50 × 6.50 m). During training with real-life stimuli (see the “Real-life” target training and the “Discrimination” training paragraphs), the T, NTO and NT were positioned on a cardboard box, which had different heights according to the dogs’ sizes (the stimuli were placed at the height of the dogs’ noses). The owners and their dogs placed themselves at one side of the room, facing the box, with the stimuli at a distance of 3 m and aligned with it. When two stimuli were presented, the cardboard boxes were placed at the same distance and at the two sides of the dogs and their owners, who were centrally positioned (see Figure S2, Supplementary Materials). An experimenter, who was the same for all subjects, placed herself behind the box, at a distance of 1.5 m and aligned with the owners and their dogs. She guided the owners through all of the training procedures and directly intervened to place the target and non-target objects (NTO and NT) on the cardboard boxes during the trials. For the digital target training and the 3D mental rotation task, digital stimuli were presented on a touchscreen that was homogeneously illuminated (Nec Multisync V321^®^ 32′′ with a refresh rate of 85 Hz and a resolution of 1280 × 1024 dpi). Both the training and the test were carried out throughout the daylight hours and the testing room was maintained under natural light conditions (no extra artificial lights were used). Before the beginning of the test, dogs with their owners stayed in the testing room for 5 min to become accustomed to the light conditions (the average brightness of the room was 197 lux). The touchscreen was placed on one side of the room at a distance of 1.5 m from the owners and their dog’s position, which was central and aligned with the screen (Figure 3). The experimenter placed herself on the right side of the screen and out of the dog’s sight. She was in charge of guiding the owner during the training and selecting all of the visual stimuli presented on the screen.

Four fixed digital video cameras were placed on tripods to record the dogs’ responses during the 3D mental rotation task (3 Sony FDR-AX43^®^, Sony Corporation, Tokyo, Japan and a GoPro 7^®^, Woodman Labs, San Mateo, CA, USA). The cameras were positioned in front of the dog and above the screen (frontal camera) to register the dogs’ spontaneous looking behaviours; on the right and the left sides of the dog–owner’s position (side cameras) to record the dogs’ reaching behaviours of the target; and behind the dog–owner dyad, facing the screen (back camera; see Figure 3).

### 2.4. Procedure

The experiment was performed in two phases, training and the test, which were carried out in sequence. In each phase, the dogs were tested individually.

#### 2.4.1. Training

The training phase aimed to teach the dog to identify a target (T) and discriminate it from the non-targets (NT and NTO). Each dog was trained to associate a specific behaviour, i.e., touching the T with its nose, with a food reward (primary reinforcer) through a shaping procedure [29]. Touching behaviour upon request was chosen as it was novel for all of the tested subjects (none of them had been trained to display it) [30]. As the tasks required the dogs to reach the target that was placed far from the owner’s position (who delivered the reward), an acoustic secondary reinforcer, i.e., the sound of a clicker, was used. This technique is known as clicker training. It is based on the combination of two forms of associative learning, i.e., classical and operant conditioning (see below for the specific training stages). When previously associated with a primary reinforcer [31], the “click-clack” sound produced by the clicker serves as a bridge stimulus between the display of the desirable behaviour and the subsequent delivery of food. Although a recent study reported no effects of the use of a clicker on dogs’ learning performances [30], the clicker was employed for practical and technical reasons, because it allowed the trainer (i.e., the owner) to instantaneously signal the behaviour to be rewarded (i.e., the touch of the T with the nose). Considering that partial rewarding (i.e., the sound is followed only 60% of the time by the food reward) is associated with negative affective states in dogs [32], continuous reinforcement schedules were used (i.e., each click sound was followed by a food reward) [33]. 

Training sessions occurred at weekly intervals at the Department of Veterinary Medicine. In each session, an average of 20 trials were conducted in which the experimenter evaluated the learning progress of each dog and supported the owners in setting the training. The owners were asked to train their dogs daily at home according to the experimenter’s instructions. The training occurred in an isolated room of the dog’s living environment and included no more than 10 trials per day (they could be split into two different sessions with an 8 h minimum interval). All dogs included in the final sample completed the training phase over an average of 25 weeks.

Training included different and consecutive stages that were completed after the fulfilment of specific learning criteria:

- Conditioning the clicker: The sound of the clicker was associated with the delivery of food (reward or primary reinforcer) through the learning process of classical conditioning [29]. Briefly, each owner presented the auditory stimulus, followed immediately by food. The dogs’ favourite food was used as a primary reinforcer, which was generally a slice of würstel [34]. After the subsequent pairing of the two stimuli, the clicker sound became a conditioned stimulus and was used as a secondary reinforcer, which then predicted the reward [31]. Each dog completed this stage within 100 repetitions of the two-stimuli pairing.
- “Real-life” target training: In this stage, the dogs were trained to identify the target by touching it with their nose (touching behaviour). The training was based on operant conditioning: when the desired behaviours were shown, dogs were rewarded with the clicker sound (secondary reinforcer), which was then followed by the delivery of food (primary reinforcer). None of the subjects spontaneously touched the target initially; instead, it was shaped through the systematic rewarding of increasingly closer approximation of the final behaviour [29,30]. Specifically, the training was sub-divided into subsequent steps: the owners held the T in their hand and presented it to their dogs at the height of their nose. In particular, the owners stood still and moved their arm towards their dogs, presenting them with the T. Intermediate steps of the complex touching behaviour were rewarded: looking at the T, approaching the T and sniffing the T. Undesired behaviours, including licking and grabbing the T, were not rewarded. Once the dogs directly touched the T with their nose in 6 consecutive trials, the target was placed close to the owners’ feet and progressively spaced from the owners’ positions until it reached the final location, i.e., on the cardboard box (3 m from the owners, see Figure S2). Each time the dogs showed the touching behaviour in 6 consecutive trials, the distance of the T from the owners’ feet was increased. An oral command “Lego” was introduced before the dogs spontaneously approached the T, as in classical conditioning. The command was then associated with the display of the desired behaviour.
- Discrimination training: Dogs were trained to discriminate the target from other non-targets. The T and NTO or NT were simultaneously placed on cardboard boxes located at a distance of 3 m from the owners’ and dogs’ positions (see Figure S2). In this stage, if the choice was correct (i.e., the dogs’ touching behaviours were directed to the T), the dogs were rewarded with the clicker sound, followed by both food and verbal rewards from the owner (“Bravo!”) in order to strengthen the positive valence of the reward. Contrarily, if the dogs touched the NTO or NT, they were not rewarded. At the end of each trial, the experimenter replaced the T and NTO or NT on the boxes. Their position was changed pseudo-randomly so that the T was placed on the same side in no more than 2 consecutive trials to avoid any side bias. Each training session always ended when the dogs made a correct choice so that they would be rewarded with a jackpot (i.e., five pieces of food) [30].

The discrimination training was sub-divided into two stages according to the non-target used. In the first stage, the dogs were trained to discriminate the T from the NTO (i.e., two different yellow plastic boxes; see Figure 1). Once the dogs reached the initial position (centrally placed with respect to the two cardboard boxes), they were given the verbal command “Lego” and spontaneously approached and possibly touched the T or NTO. Once the dogs made the correct choice by first and directly touching the T with their nose in 6 consecutive trials, they moved on to the next stage. In the second stage, the dogs were trained to discriminate between the T and NT, which only differed from each other in the position of the bricks (Figure 1). This training followed the same procedure as the first stage. It continued until the dogs met an accuracy-learning criterion of 6 correct choices over a block of 6 consecutive trials. The dogs completed the “real-life” target and discrimination training within an average of 16 weeks.

- Digital target training: the dogs learned to discriminate the digital T from the digital NT (see Figure 2), both shown at a zero view (0°). Visual stimuli were presented on the touchscreen (Figure 3). Dogs were gently led on the leash to take the initial position, i.e., facing the screen at a distance of 1.5 m and having their owners on their right side. When the dogs were centrally positioned with respect to the screen, the owners gave the verbal command “Lego” and held the leash until the dogs looked directly at the screen. The owners then left the leash and allowed the dogs to spontaneously approach and touch the T. If the dogs’ positions were not aligned with the screen, the dogs were gently led back to the initial position and the procedure was repeated. Each correct choice (i.e., touching the T with the nose) was rewarded, while incorrect choices were not. The reward procedure followed that described for the real-life and discrimination target training. The dogs were directly rewarded by their owners for each correct choice with the clicker sound followed by food and verbal reward (“Bravo!”). The automatic delivery of a food reward by the touchscreen device was avoided because, in the pilot test, the sensitivity of the screen caused some false negative responses. Each training session always ended when the dogs made a correct choice so that they were rewarded with a jackpot [30].

The digital target training was sub-divided into two stages. In the first stage, the dogs were trained to display the touching behaviour (i.e., touch the T with their nose) towards the digital target (T). This behaviour was further shaped to teach the dogs to touch the “real-life” target placed on a cardboard box close to the screen and later touch the digital T presented on the screen. Specifically, the “real-life” T was positioned ahead of the screen and centrally located. When the dogs had consistently shown a direct response towards the T in 6 consecutive trials, the “real-life” T and the box were removed and the digital T appeared on the screen (at the same position as the “real-life” T). The dogs were then asked to approach and touch the digital T with their nose. If the dogs showed any difficulties in expressing the touching behaviour (e.g., looking for the real-life T and not looking at the screen), the owners could position themselves close and oriented toward the screen to help the dogs to look at the screen and identify the T. This prompt was faded within 10 consecutive trials, in which the owners gradually reached the predetermined initial position. Once the dogs showed the touching behaviour for 6 consecutive trials, the position of the digital T was changed. The target was presented at different locations on the screen (bottom right and left, top right and left) to teach the subjects to look for and identify the T. This ability would be crucial for the next stage, where the dogs had to discriminate between the T and NT. 

In the second stage, the digital T and NT were simultaneously presented to the dogs on the two sides of the screen, evenly spaced from its centre and aligned to each other. Their position (i.e., on the left or right side of the screen) was changed pseudo-randomly, but the T was presented on the same side in no more than 2 consecutive trials to avoid any side bias. The training continued until the dogs met an accuracy-learning criterion of 6 correct choices over 6 consecutive stimuli presentations in 3 different and consecutive sessions. The dogs completed the digital target training in an average of 7 weeks.

#### 2.4.2. Three-Dimensional Mental Rotation Task

The dogs’ spontaneous abilities to recognize 3D rotated objects was explored. The testing phase consisted of two sessions with 6 trials each. In each trial, the T and NT were simultaneously presented to each dog, both having the same angle of rotation (0°, 45° or 180°). Each rotation was presented in two different trials to balance the side of the T (on the right and the left of the screen). Therefore, in each session, two trials for each angle of rotation were performed for each dog (6 trials: 2 × 3 rotation angles). The order of the rotated T and NT was changed pseudo-randomly and the stimuli with the same rotation were never shown in two consecutive trials. The testing procedure was the same as the digital training phase. During the test, the owners were asked to wear dark sunglasses in order to avoid any involuntary cues (e.g., gaze direction) provided by them that could influence the dogs’ behaviours. Each correct choice (i.e., touching of the T with the dogs’ noses) was rewarded, whereas wrong ones were not. If the dogs touched the NT first, the visual stimuli remained on the screen until they touched the T (the touching behaviour was rewarded). Therefore, each trial ended with a rewarded choice. In this way, we avoided creating any differences between rewarded (first choice correct) and non-rewarded dogs (first choice incorrect) in the trial when stimuli with the same rotation angle were subsequently presented in the same test. The testing procedure followed the same reward schedule as the training, making the procedure familiar to the test subjects. The two sessions took place with a 7-day interval. 

#### 2.4.3. Testing the Influence of the Owners’ Position

To explore the potential influence of the owners’ positions on the dogs’ potential side bias for the first choice, an additional test was performed. It took place 1 week after the main test. Two digital Ts were simultaneously presented to the dogs on the two sides of the screen, evenly spaced from its centre and aligned to each other. The test consisted of 10 consecutive repetitions of the stimuli presentation that differed only in the owners’ positions with respect to the dogs: 5 consecutive trials were performed with the owner placed to the right of the dog and the other 5 to the left side of the dog. The order of the right–left side was counterbalanced between subjects so that 3 dogs started the test with the owners on their right and the other 3 with the owners on their left. The procedure was the same as that described for the digital training phase: once the dogs reached the initial position, the owners gave the verbal command “Lego” and waited for 1–2 s until the dogs looked directly at the screen. Then, the owners left the leash and let the dog free to spontaneously approach the screen. Touching behaviour displayed to any of the Ts was rewarded.

### 2.5. Data Analysis

Due to the COVID-19 pandemic, the encounters with the experimenter, who evaluated the learning progress of dogs and supported the owners in the daily training at home, were frequently interrupted for extended periods. Unfortunately, the variability of this period’s length made it impossible for us to evaluate the individual learning curve and the number of training trials needed to fulfil the learning criteria we set. 

The dogs’ spontaneous looking behaviours towards the digital T and NT was analysed. In particular, the time spent looking at the stimulus shown on the right side of the screen and on the left one was computed (from the given command “Lego” to the first touch of any of the digital Ts or NTs). The orienting attention laterality index (LI) was then calculated with the following formula: LI = (L − R/L + R), where L indicates the total time spent looking at the digital T or NT placed on the left side of the screen and R indicates the total time spent looking at the digital T or NT placed on the right side of the screen. A score of 1.0 represents exclusively looking at the target on the left side and −1.0 represents exclusively looking at the target on the right side. A LI score of 0 indicates equal time spent looking at the T or NT placed on the right and the left side of the screen. 

Score. A binomial GLMM analysis was performed to assess the influence of “angle” (0°, 45° and 180°), “visual target side” (left vs. right), “stimulus order“, “first touch side” (i.e., the side of the screen touched first) and “rewarded side” (i.e., the side of the target that was rewarded in the previous trial) on the test variables “score” (1 = correct trial and 2 = incorrect trial), “orienting attention laterality index” and “latency to react” (i.e., the time elapsed to touch the screen), with “subjects” as a random variable. The Bayesian information criterion (BIC) was used to select and compare models based on the −2 log likelihood. To detect differences between different groups, Fisher’s least significant difference (LSD) pairwise comparisons were performed. 

Orienting attention laterality index. Given that data for the “orienting attention laterality index” were normally distributed, a GLMM analysis was performed to assess the influences of “angle” (0°, 45° and 180°), “visual target side” (left vs. right), “stimulus order“, “first touch side” (i.e., the side of the screen touched first) and “rewarded side” (i.e., the side of the target that was rewarded in the previous trial) on the test variable “orienting attention laterality index”. The Bayesian information criterion (BIC) was used for selecting and comparing models based on the −2 log likelihood. To detect differences between different groups, Fisher’s least significant difference (LSD) pairwise comparisons were performed.

Latency to react. For “latency to react” data (i.e., the time elapsed to touch the screen), survival analysis methods were used. Specifically, mixed-effects Cox regression modelling was used to analyse the influences of “angle” (0°, 45° and 180°), “visual target side” (left vs. right), “stimulus order“, “first touch side” (i.e., the side of the screen touched first) and “rewarded side” (i.e., the side of the target that was rewarded in the previous trial) on the test variable “latency to react” (with “latency to react” as the time variable and “correct trial” as the status variable). 

In addition, significant deviations from the chance level (i.e., correct trial vs. incorrect trial of the sample population at the individual level and with respect to different angles) were assessed via the one-sample Wilcoxon signed ranks test.

The inter-observer reliability was assessed by means of independent parallel coding of videotaped sessions and calculated as the percentage agreement; the percentage agreement was always more than 95% for each tested variable.

## 3. Results

**Figure 4:**
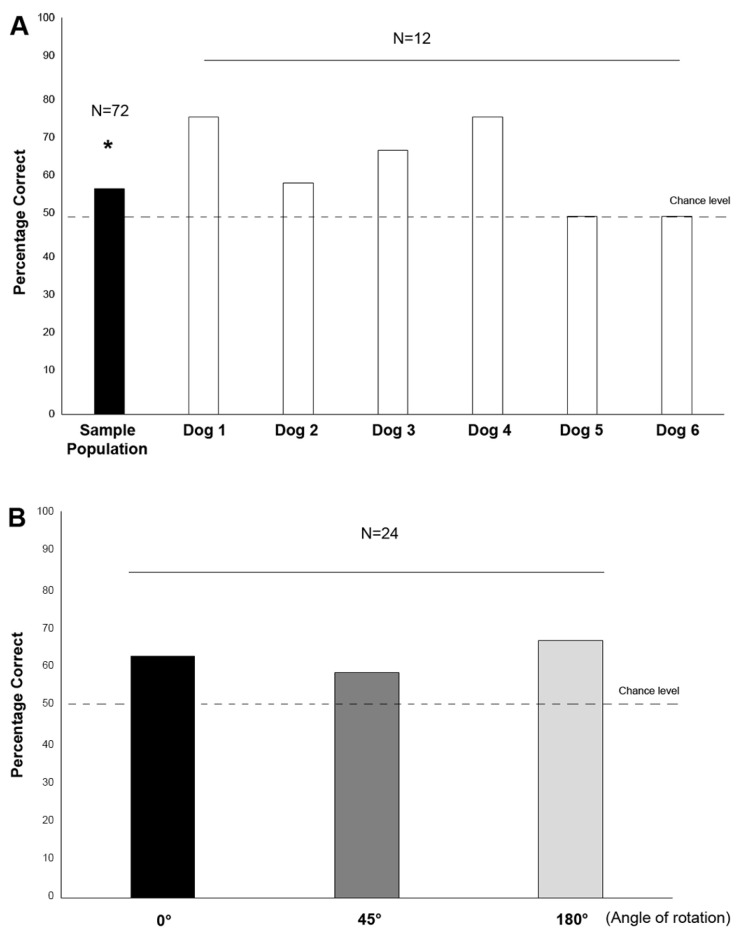
(**A**) Percentage of correct responses for the sample population and for each dog; (**B**) percentage of correct responses with respect to the different angles of rotation of the target. The black dotted line shows the chance level. * *p* < 0.05.

The results revealed that, overall, the sample population performed above the chance level (56.94% correct responses; Wilcoxon signed-ranks test, R = 1642.500, *n* = 72 trials, Z = 2.121, *p* = 0.034), even if it was not shown at the individual level (see Figure 4A; Dog 1: 75.00% correct responses, Wilcoxon’s signed-ranks test, R = 58.500, *n* = 12 trials, Z = 1.732, *p* = 0.083; Dog 2: 58.33% correct responses, Wilcoxon’s signed-ranks test, R = 45.500, *n* = 12 trials, Z = 0.577, *p* = 0.564; Dog 3: 66.66% correct responses, Wilcoxon’s signed-ranks test, R = 52.000, *n* = 12 trials, Z = 1.155, *p* = 0.248; Dog 4: 75.00% correct responses, Wilcoxon’s signed-ranks test, R = 58.500, *n* = 12 trials, Z = 1.732, *p* = 0.083; Dog 5: 50.00% correct responses, Wilcoxon’s signed-ranks test, R = 39.000, *n* = 12 trials, Z = 0.000, *p* = 1.000; Dog 6: 50.00% correct responses, Wilcoxon’s signed-ranks test, R = 39.000, *n* = 12 trials, Z = 0.000, *p* = 1.000). The percentages of correct responses with respect to the different angles of rotation of the target are shown in Figure 4B: although the percentages were above 50%, the one-sample analysis did not reveal any statistically significant differences (0°: 62.50%, Wilcoxon signed-ranks test, R = 112.500, *n* = 24 trials, Z = −1.255, *p* = 0.221; 45°: 58.33%, Wilcoxon signed-ranks test, R = 125.000, *n* = 24 trials, Z = −0.816, *p* = 0.414; 180°: 66.67%, Wilcoxon’s signed-ranks test, R = 100.000, *n* = 24 trials, Z = −1.633, *p* = 0.102).

**Figure 5:**
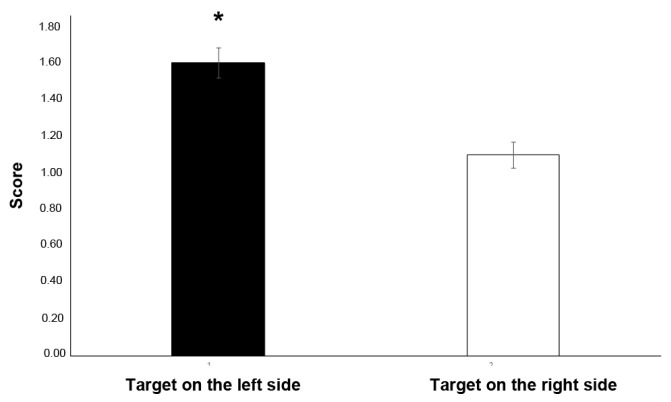
Effect of the side of presentation of the target (left vs. right side of the screen) on the score; a higher score corresponds to a higher number of correct choices (mean values of scores are presented). * *p* < 0.05.

GLMM analysis revealed a statistically significant effect of the side of the visual target on the score (F(1,49) = 8.626, *p* = 0.005, GLMM analysis; see Figure 5), indicating that the subjects performed better (i.e., had higher correct choices) when the targets were presented on the left side of the screen (right side: t35 = 0.661, *p* = 0.513; left side: t35 = −3.953, *p* = 0.000). No other statistically significant effects were revealed for “subjects” (F(5,49) = 1.133, *p* = 0.355), “angle” (F(2,49) = 1.159 *p* = 0.322), “stimulus order” (F(11,49) = 0.371, *p* = 0.961), “first touch side” (F(1,49) = 0.647, *p* = 0.425) or “rewarded side” (F(2,49) = 0.101, *p* = 0.904).

The intraclass correlation coefficient (ICC) for dog ID when it was a random effect for the “score” variable was (0.68).

**Figure 6:**
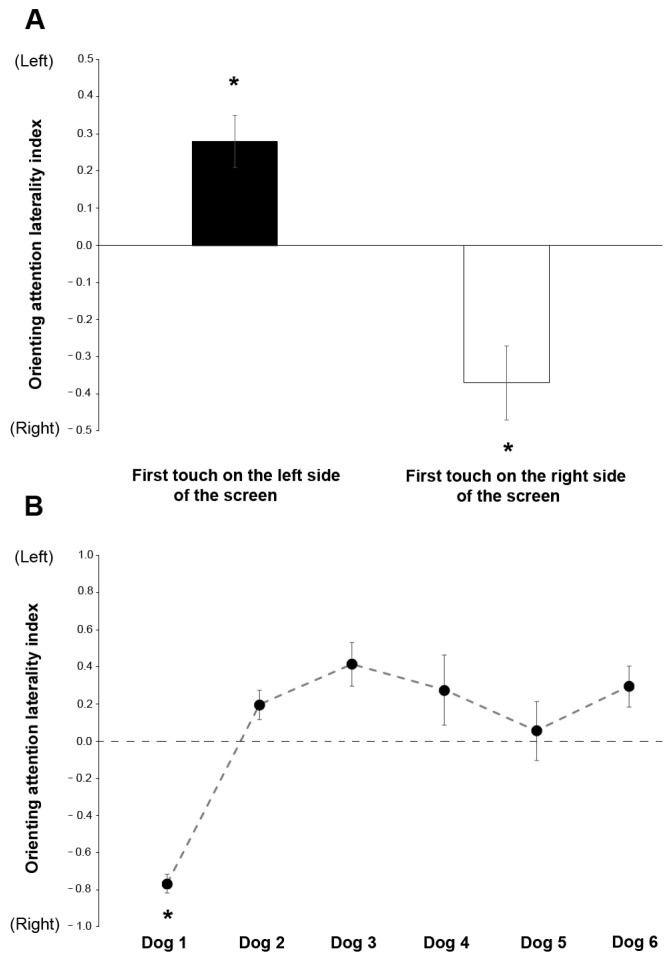
Relationship between the orienting attention laterality index and (**A**) the side of the screen touched first and (**B**) the subject dog. * *p* < 0.05.

Regarding the orienting attention laterality index, GLMM analysis revealed a statistically significant effect of the subjects (F(5,49) = 12.671, *p* = 0.000, GLMM analysis) and the side of the first touch ((F(1,49) = 31.066, *p* = 0.000, GLMM analysis); see Figure 6). Post hoc analysis revealed that Dog 1 looked more at the stimulus located on the right side of the screen than the left compared with all the other subjects (*p* < 0.001 for all comparisons). As for the side of the first touch, as expected, the post hoc analysis showed that the subjects first touched the stimulus located on the side corresponding to that which was most observed (*p* < 0.001 post hoc analysis; right side: t22 = −3.523, *p* = 0.002; left side: t48 = 3.115, *p* = 0.039). No other statistically significant effects were revealed for “angle” (F(2,49) = 0.001, *p* = 0.999); “stimulus order” (F(11,49) = 0.579, *p* = 0.837); “visual target side” (F(1,49) = 0.246, *p* = 0.622) or “rewarded side” (F(2,49) = 1.777, *p* = 0.180). The ICC for dog ID was (0.74) when it was a random effect for the “orienting attention laterality index” variable.

**Figure 7:**
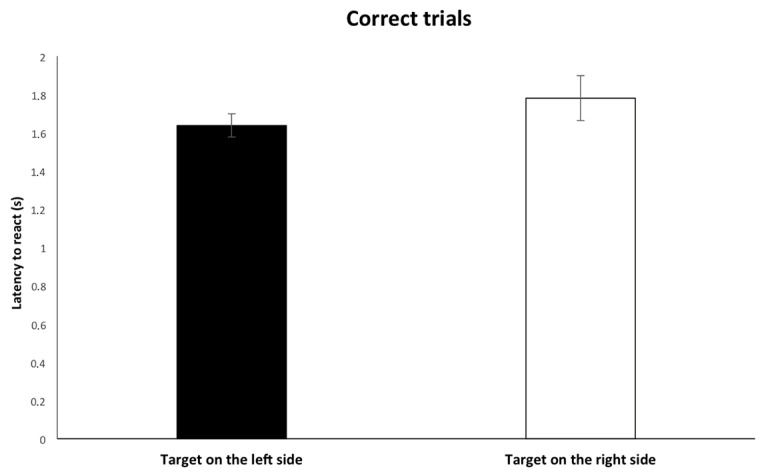
Relationship between the amount of time elapsed to touch the target (correct trial) and the side of the screen on which the target was located.

Finally, as for the latency to react, mixed-effects Cox regression revealed that the probability to react shortly to the correct target position was increased if the stimulus was presented on the left side of the screen (β(SE) = 1.57(0.41); [Exp(β) = 4.81; 95%-CI = 2.13; 10.82]; *p* = 0.000; Figure 7); no other statistically significant effects were revealed for “subjects” (β(SE) = 0.03(0.12); [Exp(β) = 1.03; 95%-CI = 0.81; 1.32]; *p* = 0.77); “stimulus order” (β(SE) = −0.09 (0.04); [Exp(β) = 0.91; 95%-CI = 0.82; 1.00]; *p* = 0.056); “first touch side” (β(SE) = −0.37(0.39); [Exp(β) = 0.68; 95%-CI = 0.31; 1.48]; *p* = 0.34) or “rewarded side” (β(SE) = 0.39(0.49); [Exp(β) = 1.48; 95%-CI = 0.56; 3.87]; *p* = 0.42).

The ICC for dog ID was (0.67) when it was a random effect for the “the latency to react” variable.

GLMM analysis did not show any effects of the owner’s position on the side of the screen touched first (F(1,5) = 1.943, *p* = 0.222).

## 4. Discussion

Overall, our results showed that dogs recognized three-dimensional objects and their rotated versions more easily when presented on the left side of the screen. This result indirectly indicates a dominant role of the right hemisphere in visuo-spatial abilities [35]. The evidence for right-hemispheric specialization in spatial processing has been reported in several human and non-human species. In rats, for example, Bianki [36] showed that the visual discrimination of a series of stimuli presented simultaneously in the left and right visual hemifields was impaired by the inactivation of the right hemisphere, but not the left. In a similar way, Bradshaw and Rogers [37] found that male rats’ abilities to solve mazes were poorer when cycloheximide, which temporarily inhibits protein synthesis, was injected into the right hemisphere than when it was injected into the left. In chicks, a prevalent role of the right hemisphere has been reported in both handling geometric information and in controlling spatial attention resources [38]. The latter has also been reported in neuropsychological studies in humans, such as cancellation tasks, providing additional evidence of the prevalent role of the right hemisphere in spatial attention [39]. However, conflicting results have been reported from studies on primates. In prosimians, a left-handed preference in visually guided movements has been reported, suggesting the prevalent role of the right hemisphere in spatial tasks [40]. On the other hand, split-brained monkeys, for example, displayed a left-hemispheric superiority in discriminating lines with varying orientations [41]. Similarly, in rhesus monkeys, a left-hemispheric advantage in localizing a dot in a square surround was revealed [42]. In canine species, the use of the right hemisphere for spatial abilities has been previously reported during performances in agility-trained dogs [43]. Specifically, the latency to perform agility obstacles was longer when an arousing stimulus (i.e., the owner) was located in the left visual hemifield (right hemisphere use), rather than the right. It was also related to the spatial complexity of the task, demonstrating that the presence of an overload of information in the right hemisphere is simultaneously involved in the processing of higher emotional stimuli and spatial information. Here, the right hemisphere advantage for processing the visuo-spatial information could also be suggested by the shorter reaction time registered when the 3D objects were located on the left side of the screen, i.e., in the left side of the dogs’ visual spaces. Indeed, a relationship between right hemisphere activity and shorter latency to react to sensory information was previously found in dogs for visual [44] and auditory stimuli [45].

In a very recent study using 2D visual stimuli, it was reported that dogs were capable of solving a task similar to those that humans solve with mental rotation [23]. Specifically, eight dogs were trained to discriminate between bidimensional shapes presented on a computer screen. The dogs’ accuracies and head tilts during testing with rotated versions of the same shapes were measured. Although the overall accuracy of the sample population generalization to rotated stimuli was 55%, three dogs of eight performed differently from the chance level. The amplitude of stimulus rotation did not influence the dogs’ performances, as described in humans and in sea lions [9], suggesting the absence of a mental rotation process in canine species. Our results support this hypothesis, because we found that the angle of rotation of the target did not affect the dogs’ performances (i.e., correct choices) or their reaction time. It could be possible, therefore, that dogs use a rotational invariance process for the discrimination of rotated shapes, as previously reported in pigeons [2], which could mainly occur in the right hemisphere. However, further studies examining the animals’ responses to 3D mirrored pictures are needed to confirm this hypothesis.

Similar to Lonardo and colleagues [23], we found that the sample population performed above the chance level (56.94% correct responses), but this result was not confirmed at the individual level, suggesting that the recognition of 3D rotated objects requires complex cognitive abilities that may challenge dogs. Moreover, although dogs performed the test when they fulfilled the learning criteria of 100% of correct choices during training, we registered a significant decrease in performance (62.50%) for the recognition of the target at 0 degrees during the test. This result could be explained by the increasing complexity of the task, in which, contrarily to the training, where only the side of the target presentation changed, in each trial, the spatial orientation of the target was different.

We report inter-individual variability in the performance of dogs in the visuo-spatial task. Specifically, we found that both the latency to react and the orienting attention laterality index, which indirectly indicate the different activation of the two brain hemispheres, significantly varied between subjects. Several factors contribute to the strategy used to solve visuo-spatial cognitive tasks and may explain the differences we registered: the use of local or global information to process the stimuli [15], the motivation to complete the task (and to obtain food) [23], which may account for the differences in the subjects’ reaction times found here, and the individual spatial abilities [17,18]. Contrary to humans [18,19], we found that the level of practice in mental rotation tasks did not affect the performance of each individual because no differences in dogs’ responses were found according to the repetition of stimuli presentation. Given the small number of subjects involved in the experiment, the influence of sex on the dogs’ performances was not tested. However, although gender plays an important role in spatial ability in humans [16,17], a previous study reporting no differences between male and female dogs in bi-dimensional rotational tasks (for the recognition of rotated objects) suggests that an influence of sex on dogs’ spatial abilities is less likely [23]. On the other hand, the preferential use of a paw or a hand [5,17,20,46] is significantly related to visuo-spatial ability in both humans and dogs. Therefore, it would certainly be of interest in future studies to also consider the possible relationship between motor laterality and the ability to recognize three-dimensional rotated images, given that, in dogs, there is evidence of a relationship between the preferential use of the front paws in a motor task and visuospatial attention [4]. Individual differences were also registered in the orienting attention laterality index indicating that Dog 1 consistently oriented its visuo-spatial attention to the right side of the screen. The lack of a significant bias at the individual level for the other dogs was unexpected. Although there is considerable evidence of right specialization for spatial abilities [17,18], some authors report that the three-dimensional rotation of Shepard–Metzler shapes may require the left hemisphere’s involvement, which increases with the complexity of the task [17]. The left hemisphere contributes to the part-wise decomposition of shapes and sequential organization, as reported by Corballis [47]. It could be possible, therefore, that the complementary introduction of left-hemispheric components could have decreased the difference between the activation of the hemispheres, which could explain the lack of significant bias observed at the individual level. However, further studies with a higher number of trials for each individual are needed to support this hypothesis and to shed light on the potential factors influencing the attention-orienting bias.

In humans, EEG and lesion studies analysing the mental rotation of 3D objects report the activation of both the right and the left hemispheres participating in different subcomponents comprising mental rotation [3,18]. Specifically, the right hemisphere mediates encoding and comparison/decision processes, while the left hemisphere is involved in the generation of images, decomposition of shapes, sequential organization and object mental rotation. The parallel processing of the rotated 3D objects by the right and the left hemisphere, whose main involvement could vary among individuals, may, therefore, explain the absence of a significant bias registered at the individual level.

## 5. Conclusions

Although our preliminary results showed that dogs had the potential ability to recognize three-dimensional rotated images and that this ability was predominantly under the control of the right cerebral hemisphere, future studies on a larger sample population are needed before generalizing these findings.

## Data Availability

The data presented in this study are available in the Supplementary Materials.

## Supplementary Materials

The following supporting information can be downloaded at: https://www.mdpi.com/article/10.3390/ani13101673/s1, Figure S1: Digital realistic representation of the target (zero view, 0°) with the coordinate system used; Figure S2: Experimental setup of the “real-life” target and discrimination training; Dataset.
