# Supplementary material for: Effect of Attentional Bias on the 3D Rotated Objects Recognition Ability of Dogs
Source: Animals (Basel). 2023 May 17;13(10):1673. doi: 10.3390/ani13101673 (PMC10215826; doi:10.3390/ani13101673)
Supplement: Supplementary file 1 [file animals-13-01673-s001.zip › Supplementary materials.pdf]

Effect of attentional bias on the 3D rotated objects recognition ability in dogs

Siniscalchi Marcello, d'Ingeo Serenella*, Quaranta Angelo

[1]Animal Physiology and Behaviour Unit, Department of Veterinary Medicine, University of Bari Aldo Moro, 70121 Bari, Italy.

*email: serenella.dingeo@uniba.it

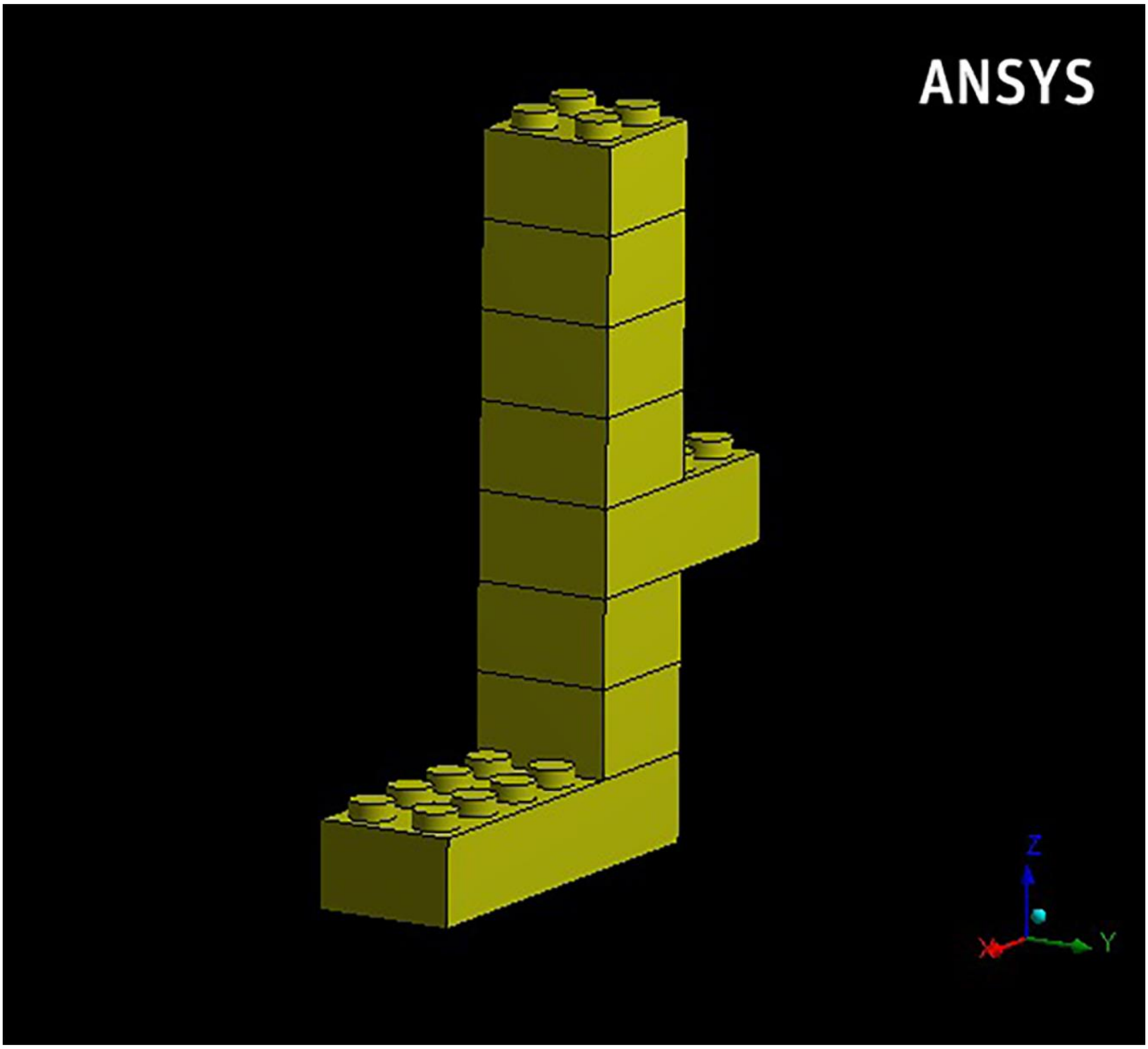

Figure S1. The digital realistic representation of the target (zero view, 0°) with the coordinate system used.

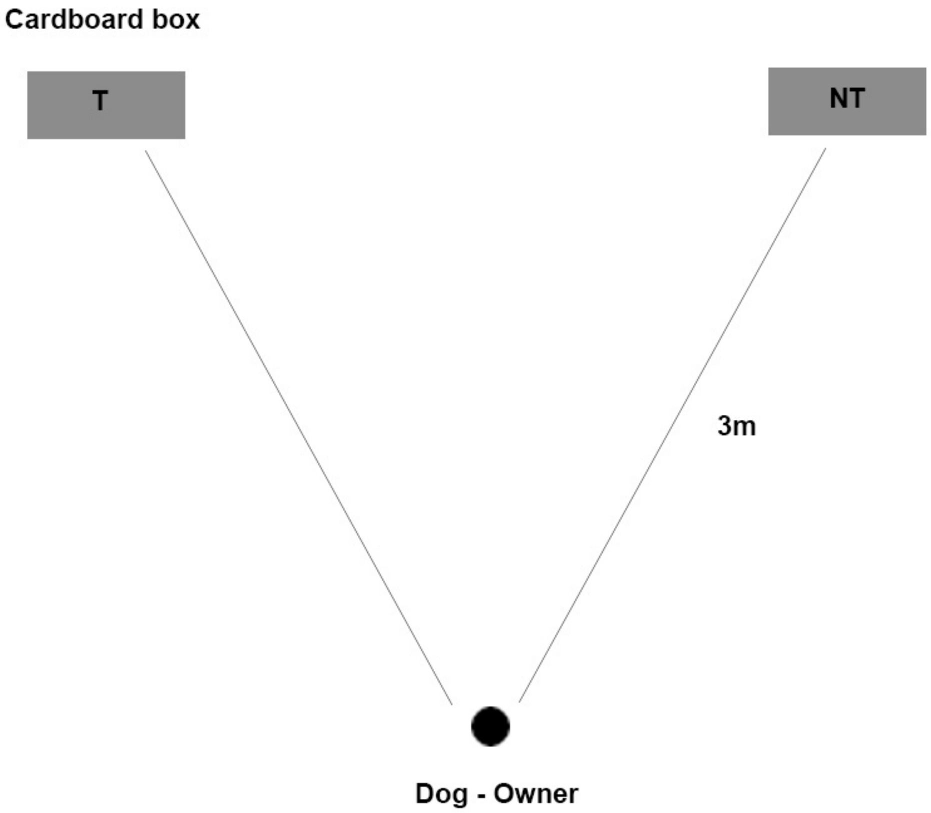

Figure S2. Experimental setup of the “real-life” target and discrimination training
